# Histamine intolerance and anxiety disorders: pilot cross-sectional study of histamine intolerance prevalence in cohort of patients with anxiety disorders

**DOI:** 10.1192/j.eurpsy.2022.980

**Published:** 2022-09-01

**Authors:** E. Nosková, K. Vochosková, V. Knop, P. Stopková, M. Kopeček

**Affiliations:** 1 Charles University in Prague, Third Faculty Of Medicine, Prague, Czech Republic; 2 National Institute of Mental Health, Medical Care, Klecany, Czech Republic

**Keywords:** anxiety symptoms, diaminooxidase, genetic examination, histamine intolerance

## Abstract

**Introduction:**

Histamine intolerance (HI) is a disorder associated with an impairment of ability to metabolize ingested histamine. The incidence of HI in general population is 1-3%. Clinical manifestation of HI contains nonspecific predominantly gastrointestinal, but also extraintestinal symptoms. HI could be primary with genetic predisposition, or secondary with lower activity of diaminooxidase (DAO) without positive genetic screening.

**Objectives:**

This study aims to evaluate the prevalence of HI by patients with anxiety disorders. HI can imitate anxiety symptoms, therefore we predict higher prevalence HI in patients with anxiety disorders than in general population.

**Methods:**

It is observational cross-sectional study on cohort of anxious patients for detecting the prevalence of HI. Patients were screened by scale for histamine intolerance questionnaire. Patients with positive questionnaire were examined for serum DAO and genetically examined.

**Results:**

113 patients fulfilled the HI questionnaire. From this cohort 35.4% (40 subjects) were positive at screening. Biomarkers of HI were screened only in case of positivity in this questionnaire. **Table No. 1:** Results of our study from cohort with positive screening, 35.4 % (40 subjects).

**Table:** 

		**Genetic predisposition**		
		**positivity in risk allele**	**negativity in risk allele**	**altogether**
**Serum level of DAO**	**positive screening** (DAO<10 U/ml)	**5 (4.4%)** *primary-genetically determined HI*	**5 (4.4%)** *secondary HI*	10
	**negative screening** (DAO≥10 U/ml)	14 (12.4%)	16 (14.2%)	30
	altogether	19	21

**Conclusions:**

This pilot study shows that the prevalence of HI could be higher in group of patients with anxiety disorders than in general population. For further confirmation other studies with control group and larger cohort should be done.

**Disclosure:**

No significant relationships.

